# The Turkish validation of the Brief International Cognitive Assessment for Multiple Sclerosis (BICAMS) battery

**DOI:** 10.1186/s12883-017-0993-0

**Published:** 2017-12-06

**Authors:** Serkan Ozakbas, Pinar Yigit, Bilge Piri Cinar, Hatice Limoncu, Turhan Kahraman, Görkem Kösehasanoğulları

**Affiliations:** 1 Department of Neurology, Dokuz Eylul University, Izmir, Turkey; 2 Department of Neurology, Samsun Training and Researce Hospital, Samsun, Turkey; 3 School of Physical Therapy and Rehabilitation Department, İzmir Katip Celebi University, Izmir, Turkey; 4 Department of Neurology, Usak State Hospital, Usak, Turkey

**Keywords:** Multiple sclerosis, BICAMS battery, Cognitive impairment

## Abstract

**Background:**

Cognitive impairment may be seen in as many as 43–70% of patients with multiple sclerosis (MS) and may be observed in all MS subtypes. The Brief International Cognitive Assessment in Multiple Sclerosis (BICAMS) battery may be used to evaluate cognition status. The purpose of the current study is to validate the BICAMS battery in Turkish.

**Methods:**

Patients with MS attending our clinic between September 2014 and April 2015 were invited to participate. Healthy control participants were matched in terms of age, gender and years of education.

**Results:**

One hundred seventy-three MS patients and 153 healthy control participants were enrolled in the study. MS patients performed significantly worse in all trials than the members of the healthy control group. In addition, cognitive dysfunction was identified in 78 of the 173 (45.1%) patients. In the MS with cognitive impairment group, 64 out of 151 (42.4%) subjects were RRMS patients, 12 out of 18 (66.7%) were secondary progressive MS patients, and 2 out of 4 (50%) were primer progressive MS patients.

**Conclusions:**

The BICAMS has been proposed for assessing cognitive impairment in MS patients. This study shows that the battery is suitable for use in Turkey.

## Background

Cognitive impairment is common in multiple sclerosis (MS), and approximately half of patients with MS present with cognitive impairment that adversely impacts on aspects of both patients’ and caregivers’ everyday lives [1, 2]. It is demonstrable in all disease stages and subtypes, in up to 40% of newly diagnosed individuals with clinically isolated syndrome and relapsing remitting MS (RRMS) [3] and in up to 60% of those with secondary progressive MS (SPMS) [4]. It can have a significant impact on quality of life and can influence employment status, physical independence, communications, treatment adherence and even rehabilitation benefit [2]. The assessment of MS-related cognitive decline has received increasing attention in recent decades. Many different neuropsychological batteries have been proposed. However, the Brief Repeatable Battery of Neuropsychological tests (BRB-N) [5] and the Minimal Assessment of Cognitive Function in MS (MACFIMS) [4, 6] are the most popular tools. While both batteries are known to be highly specific for the evaluation of cognitive impairment in MS patients, their implementation in everyday clinical practice remains limited due to their high time demands (at least 45 min are required for BRB-N and 90 min for MACFIMS) and the need for surveillance and interpretation by specialist neuropsychologists [4–7]. Various neuropsychological batteries have been proposed for the assessment of cognitive impairment in MS as the interest in this area has increased over recent years. The Brief International Cognitive Assessment in Multiple Sclerosis (BICAMS) was proposed by an expert panel as a tool for brief cognitive monitoring of MS patients in clinical settings in 2012 [8]. It can be administered by healthcare professionals without any formal neuropsychological training to identify early or subtle cognitive impairment. The BICAMS battery is a fast, reliable, sensitive and specific tool that has been validated and applied in many countries [9–14]. The primary objective of our study was the cross-cultural validation of the BICAMS battery to Turkish. The secondary objective was to measure the impact of cognitive impairment on patients’ quality of life and the effect of fatigue on patients’ cognitive state by assessing correlations between BICAMS performance and the Modified Fatigue Impact Scale (MFIS) and the Multiple Sclerosis International Quality of Life (MUSIQoL) questionnaire.

## Methods

### Patients

Patients with a diagnosis of MS according to the 2010 revised McDonald criteria [15] attending our clinic between September, 2014, and April, 2015, were invited to participate. Patients were recruited cross-sectionally, and no pre-selection was applied for cognitive impairment. The inclusion criteria were age over 18 years, the ability to give informed consent, neurological stability with no evidence of relapse, being steroid and/or plasmapheresis-free for at least 4 weeks preceding enrollment, and proficiency in the Turkish language. Patients were excluded if they had a current or previous neurological disorder other than MS, a current psychiatric disorder unrelated to that diagnosis, a coexistent medical condition that might influence cognition, a previous history of developmental disorder unrelated to MS, a history of learning disability, any vision or hearing problems that might influence performance on the tests, or a current or past history of alcohol or drug abuse. Control participants were recruited from unaffected relatives or friends of MS patients or from other individuals attending the neurology outpatient clinic for other reasons, such as migraine or vertigo. All relatives were matched in terms of age, gender and years of education. All patients and all healthy control subjects provided verbal informed consent to participation in the study. Approval for the research project was granted by the Ethics Committee of Dokuz Eylul University of Izmir.

### Study instruments and procedures

The methodology employed followed the recommendations for BICAMS national validation (step 1; standardization and translation of test stimuli, step 2; standardization and translation of test instructions, step 3; normalization, step 4; test-retest reliability, step 5; criterion-related validity) [16]. Age, sex, handedness, years of education, occupation and employment status were recorded for all participants. In the MS group, disease subtype, expanded disability status scale (EDSS) [17] and disease duration from onset of symptoms were also noted. Depression was assessed using the Beck Depression Inventory (BID) [18], and the (MFIS) [19] was used to measure fatigue.

All participants were administered the BICAMS, and test re-test reliability was confirmed for all patients and controls. The BICAMS is composed of the Symbol Digit Modalities Test (SDMT), California Verbal Learning Test (CVLT-II) and Brief Visuospatial Memory Test Revised (BVMTR) [8]. The SDMT measures the working memory and information processing speed. It consists of nine symbols, each representing a number from 1 to 9. These pairs are visible in a key at the top of an A4-size page. Below are a number of rows of the same symbols arranged in random order. After a short practice session, subjects are asked to match as many of the symbols with the digits as they can in 90 s. The answers can be written or orally. We used the oral version, and number of correct answers recorded within 90 s [20].

The CVLT-II is composed of a list of 16 words in 4 semantic categories. The examiner reads out the list of words at a steady pace of in approximately 20 s. The patient listens to the complete list and is then asked to repeat as many of the words as possible, in any order, which the examiner records on a piece of paper. Five trials in total are performed, and the final score is composed of the total number of words recorded across these trials. On each occasion the patient is asked to remember the answers given in the preceding trial. The BVMT-R consists of 6 abstract symbols on an A4-size page. Patients are given 10 s to look at the page, which is then removed from view. They are then asked to draw as many symbols as they can remember in the order given on a blank page. These symbols are then scored from 0 to 2, depending on accuracy and location. This is performed 3 times, and the total score consists of the sum of scores from all 3 trials.

Quality of life (QoL) was assessed using the MUSIQoL questionnaire [21]. This consists of 31 questions in 9 dimensions (subscales): activities of daily living (8 items), psychological well-being (4), symptoms (4), relationships with friends (3), relationships with family (3), sentimental and sexual life (2), coping (2), rejection (2), and relationships with the healthcare system (3). The index score is computed as the mean of these subscale scores. We used only the index score, which was linearly transformed and standardized on a 0 to 100 scale, where 0 indicates the worst possible level of QoL and 100 indicates the best possible level. The MFIS battery consists of 40 questions (3 subscales; social functions, physical functions and cognitive functions). Each question is scored from 0 (minimal problems) to 4 (severe) [19].

### Statistical analysis

Comparisons between groups were performed using paired-sample t-tests and the Mann–Whitney U test for continuous variables where appropriate. The chi square test was used for categorical variables. Normal distribution of data was verified using the Shapiro-Wilk test. Multinomial logistic regression was performed to evaluate associations among the individual neuropsychological tests, and depression, education status, disability level, duration of the disease, and the relapse rate. Correlation of BICAMS test-retest scores was evaluated using Pearson correlation coefficient r. Statistical analysis was performed on SPSS 15.0 software. Statistical significance was set at *p* < 0.05.

## Results

One hundred seventy-three MS patients and 153 healthy controls were recruited to the study. Baseline characteristics are outlined in Table 1. Of the MS patient group, 151 (87.3%) were classified as having RRMS, 18 (10.4%) with SPMS and 4 (2.3%) with primary progressive MS (PPMS). Mean EDSS scores were 2.1 (SD: 1.1), 4.6 (SD: 1.5) and 5.3 (SD: 1.6), respectively, with disease durations of 7.1 (SD: 5.6), 14.9 (SD: 9.1) and 11.5 (SD: 8.4) years. Table 2 shows the group data and the differences between the patient and healthy groups’raw scores in all 3 trials and during the retests. The MS patients performed significantly worse in all trials than the members of the healthy group. As no validated threshold of cognitive impairment for BICAMS was available, we identified the 5th percentile on each performance of the healthy group in order to evaluate how many MS patients were impaired on each of the three components of BICAMS. Based on the proposed criterion of one or more test performances being below the 5th percentile of the healthy controls’ performance, we identified cognitive dysfunction in 78 of the 173 (45.1%) patients. Of the MS with cognitive impairment group, 64 out of 151 (42.4%) subjects were RRMS patients, 12 out of 18 (66.7%) were SPMS patients, and 2 out of 4 (50%) were PPMS patients. When the MS group was further subdivided into RRMS and progressive MS (SPMS and PPMS) groups, statistically significant differences were observed between the groups in each of the individual tests (p˂0.05). Using the cut-off of one or more impaired tests, 41.7% of the RRMS and 77.3% of the progressive MS patients met the criterion for cognitive impairment. Table 3 summarizes the results and the estimation of impaired MS patients on one, two, and three tests.

**Table 1:** Baseline characteristics of patient and control participants

	MS Patients	Control Group	P
Gender n (%)			
Female	124 (71.7)	109 (71.2)	NS
Male	49 (28.3)	44 (28.8)	
Age (years) mean ± SD	37.5 ± 10.7	36.9 ± 8.9	NS
Education (years) mean ± SD	13.9 ± 7.3	15.4 ± 8.8	NS
Employment n (%)			
Employed	41 (23.7)	59 (39.1)	0.008
Unemployment or not working due to MS	45 (26)	20 (13.2)	0.024
Not working by choice/housewife/retired	71 (39.7)	58 (38.4)	NS
Student	16 (9.2)	14 (9.3)	NS
Disease duration (years), mean ± SD	9.2 ± 6.1	–	–
EDSS score, mean ± SD	2.4 ± 1.7	–	–

*NS* not significant, *SD* standard deviation, *EDSS* expanded disability status scale

**Table 2:** Differences between the patient and healthy groups’raw scores in all 3 trials and during the retests

	Raw Score (SD)		
	MS Patients	Healthy Controls	p
SDMT	43.2 (12.5)	53.5 (9.5)	<0.001
SDMT retest	46.6 (16.4)	56.1 (10.2)	<0.001
CVLT-II	45.7 (11.3)	53.9 (7.7)	0.002
CVLT-II retest	47.9 (16.2)	60.2 (9.2)	0.003
BVMT-R	16.9 (8.5)	22.5 (9.2)	0.002

**Table 3:** Prevalence of cognitive impairment in MS patients according to the 5th percentile value of HC on BICAMS tests

	RRMS n (%)	SPMS n (%)	PPMS n (%)	Total)
SDMT	56 (37.1)	13 (72)	2 (50)	71 (41)
SDMT retest	55 (36.4)	13 (72)	2 (50)	70 (40.5)
CVLT-II	54 (35.7)	11 (60.6)	1 (25)	66 (38.2)
CVLT-II retest	56 (37.1)	10 (55.6)	1 (25)	67 (38.7)
BVMT-R	48 (31.8)	9 (50)	1 (25)	58 (33.5)
BVMT-R retest	46 (30.5)	10 (55.6)	1 (25)	57 (32.9)
On 1 test	63 (41.7)	14 (77.8)	3 (75)	80 (46.2)
On 2 tests	35 (23.2)	8 (44.4)	1 (25)	44 (25.4)
On 3 tests	21 (13.9)	5 (27.8)	1 (25)	27 (15.6)

*SDMT* symbol digit modalities test, *CVLT-II* California verbal learning test II, *BVMT-R* the brief visuospatial memory test revised

When the patient group was subdivided in terms of disease duration, 36.6% of those diagnosed < 10 years previously (45 out 14 of 123) and 64% diagnosed ≥10 years (32 out of 50) previously also met the criterion for cognitive impairment (data not shown). Significant negative correlations were determined between SDMT scores and EDSS (*r* = −0.46, *p* = 0.003), and between CVLT II 17 scores and EDSS (r = −0.4, *p* = 0.024). No significant correlation was determined with BVMT-R (*r* = −0.24, *p* > 0.05). As expected, higher rates of unemployment were seen amongst the patient population compared to the control participants. We also determined higher rates of unemployment or inability to work due to MS in patients with cognitive impairment (34 vs 11 patients, respectively, p0.05). The test-retest interval was calculated as a median value of 15 days, with a mean value of 14.2 ± 4 days (10–21 days). We evaluated the difference between the patient and the healthy group test-retest scores (Table 4). Table 4 shows that the r values in the patient group were higher than those of the healthy controls (*r* = 0,814 vs r = 0,714).

**Table 4:** Correlation coefficients between the tests and the retests

	Overall		Patient		Healthy controls	
	R	P	R	P	R	P
SDMT	0.814	<0.001	0.862	<0.001	0.714	<0.001
CVLT-II	0.892	<0.001	0.904	<0.001	0.792	<0.001
BVMT-R	0.828	<0.001	0.869	<0.001	0.706	<0.001

*SDMT* symbol digit modalities test, *CVLT-II* California verbal learning test II, *BVMT-R* the brief visuospatial memory test revised

In order assess the impact of fatigue on patients’ cognitive status, we examined the correlations between their scores during the BICAMS battery and the MFIS battery scores. We determined significant negative correlations between the patients’ overall subjective fatigue scores and their cognitive performance in all parts of the BICAMS (Table 5). The decline in cognitive functions measured by the BICAMS correlated best with the cognitive dimension subscale of the MFIS, followed by the physical subscale. We only determined meaningful correlation between the SDMT and the social subscale of the MFIS. As shown in Table 6, cognitive impairment correlated significantly with all MUSIQoL subscales. The most prominent correlations were with “activities of daily living”, “psychological well-being” and “sentimental and sexual life”.

**Table 5:** Correlations between the FIS battery and its subscales with the parts of the BICAMS battery

	Total		Cognitive		Physical		Social	
	R	P	r	P	r	p	R	p
SDMT	−0.428	<0.001	−0.463	<0.001	−0.386	0.002	−0.302	0.046
CVLT-II	−0.382	0.003	−0.414	0.004	−0.362	0.021	−0.268	0.076
BVMT-R	−0.316	0.026	−0.396	0.034	−0.298	0.045	−0.245	0.081

*SDMT* symbol digit modalities test, *CVLT-II* California verbal learning test II, *BVMT-R* the brief visuospatial memory test revised

**Table 6:** Correlations between performance in BICAMS battery and score in MUSIQoL battery

	SDMT		CVLT-II		BVMT-R	
	r	p	r	p	r	p
Activities of daily living	0.445	<0.001	0.412	<0.001	0.403	<0.001
Psychological well-being	0.489	<0.001	0.398	<0.001	0.387	<0.001
Symptoms	0.402	0.004	0.378	0.008	0.335	0.012
Relationships with friends	0.389	0.014	0.316	0.024	0.372	0.014
Relationships with family	0.394	0.002	0.332	0.009	0.368	0.008
Sentimental and sexual life	0.512	<0.001	0.438	<0.001	0.475	<0.001
Coping	0.342	0.023	0.316	0.044	0.325	0.018
Rejection	0.358	0.028	0.318	0.041	0.340	0.007
Relationships with the healthcare system	0.306	0.042	0.322	0.048	0.314	0.04

*SDMT* symbol digit modalities test, *CVLT-II* California verbal learning test II, *BVMT-R* the brief visuospatial memory test revised

## Discussion

The assessment of cognitive status and characterization of damaged domains are useful for all MS patients in terms of appropriate rehabilitation, vocational counseling, and quantification of disability, beginning from the initial stages of the disease. It is now recognized that assessments and follow-ups should be as much as a priority as the evaluation of physical disability. Many diagnostic batteries have been used for these purposes. The most commonly used batteries of neuropsychological tests in MS are the BRB-N and MACFIMS, which are accurate, but may be time-consuming to administer. These batteries are not, therefore, suitable for everyday clinical practice, especially in centers without a resident neuropsychologist.

There is a need for a brief cognitive assessment tool with adequate reliability, validity, specificity and sensitivity, capable of provide comprehensive and accurate cognitive evaluations. The BICAMS has been proposed for assessing cognitive impairment in MS patients [8, 16]. The BICAMS battery can be used in small MS centers without a resident neuropsychologist [22]. Translation and validation studies of the BICAMS battery are currently being performed in several countries.

In our study, we assessed the cognitive status of MS patients using the Turkish translation of the BICAMS and measured the impact of cognitive impairment on the demographic and clinical characteristics. In our validation process, we observed significant differences in all tests between the MS group and the healthy group. The most significant difference was determined in the SDMT. We also determined strong correlations (*p* < 0.001) when assessing the test-retest reliability in both the patient and HC groups. Correlations were stronger in the patient group (*r* > 0.8) than in the HC group (r between 0.7 and 0.8).

In agreement with many other studies [3, 23], level of disability and duration of disease were associated with severity of cognitive impairment in the present research. However, some studies have also reported that duration of disease was not correlated with cognitive impairment [10]. Unemployment or inability to work due to MS was also correlated with cognitive impairment. Although patients often report that fatigue impairs their cognitive abilities [24], the majority of studies have failed to identify any correlation between fatigue and cognitive involvement [25–29]. But, fatigue has been reported to be capable of an adverse impact on patients’ cognition [12, 30]. We observed significant negative correlations between patients’ overall subjective fatigue scores and their cognitive performance in all parts of the BICAMS. Sandi et al. reported that all FIS scores exhibited meaningful negative correlations with patients’ BICAMS performances. The strongest correlation was determined in SDMT [12]. In terms of the FIS subscales, they observed that the physical subscale exhibited the strongest correlation with cognitive status, while the cognitive subscale exhibited meaningful correlation both the SDMT and the CVLT-II tests. In our study, however, the decline in cognitive functions measured using BICAMS correlated best with the cognitive dimension subscale of the FIS, followed by the physical subscale. The SDMT was only significantly correlated with the FIS social subscale.

Several studies have reported that a decline in cognitive status is associated with poorer QoL [12, 31, 32]. Our study yielded similar results. All MUSIQoL subscales exhibited significant correlation with scores on the BICAMS battery. The most prominent correlations were with “activities of daily living”, “psychological well-being” and “sentimental and sexual life”. Sexual life is the subscalemost commonly correlated with cognitive status in ours and previous studies [12, 31–34].

The BICAMS is useful as a monitoring test for identifying MS patients with cognitive impairment. The Turkish version of the BICAMS is a short, easily administered, and specific tool for the clinical evaluation of cognitive impairment in MS patients, and is as reliable as the original English version. However, there are a number of limitations to our study. Some of the healthy control group being relatives of MS patients may be one such limitation, because there is a known greater incidence of MS in the relatives of MS patients. However, no significant difference was determined between this control subgroup and the other healthy control group in any tests. Moreover, alternative forms were used for the BVMT-R and SDMT testretest applications in order to avoid the practice effect. However, since there is only one CVLTII form, that single form was used. This may have led to a practice effect for CVLTII, and may constitute a limitation.

## Conclusions

The present study contained the highest number of patients and healthy subjects to date. We want to emphasize that fatigue can have a negative effect on patients’ cognitive status, while cognitive impairment can impact on employment status among patients with MS.

## Abbreviations

BICAMS: Brief International Cognitive Assessment In Multiple Sclerosis
BID: Beck depression ınventory
BRB-N: Brief repeatable battery of neuropsychological test
BVMTR: Brief visuospatial memory test revised
CVLT-II: California verbal learning test
EDSS: Expanded disability status scale
MACFIMS: Minimal assessment of cognitive function in multiple sclserosis
MFIS: Modified fatigue ımpact scale
MS: Multiple sclerosis
MUSIQoL: Multiple sclerosis ınternational quality of life
PPMS: Primary progressive multiple sclerosis
RRMS: Relapsing remitting multiple sclerosis
SDMT: Symbol digit modalities test
SPMS: Secondary progressive multiple sclerosis
